# Urine Galectin-3 binding protein reflects nephritis activity in systemic lupus erythematosus

**DOI:** 10.1177/09612033221145534

**Published:** 2022-12-12

**Authors:** Francesca Faustini, Helena Idborg, Enrico Fuzzi, Anders Larsson, Wen-Rong Lie, Sven Pötzsch, Shinji L Okitsu, Elisabet Svenungsson, Iva Gunnarsson

**Affiliations:** 1 Division of Rheumatology, Department of Medicine Solna, Karolinska University Hospital, 27106 Karolinska Institute, Stockholm, Sweden; 2 Division of Rheumatology, Department of Medicine DIMED, Padua University Hospital, Padua, Italy; 3 Department of Medical Sciences/Clinical Chemistry, 8097 Uppsala University, Uppsala, Sweden; 4 EMD Millipore Corporation, St Louis, MO, USA; 5 2792 Merck KGaA, Darmstadt, Germany; 6 189697 EMD Serono Research and Development Institute, Billerica, MA, USA

**Keywords:** Systemic lupus erythematous, lupus nephritis, urinary biomarkers, Galectin-3 binding protein

## Abstract

**Background:**

Lupus nephritis (LN) is a major and severe organ involvement in systemic lupus erythematosus (SLE), whose diagnosis and treatment necessitate to perform kidney biopsy, which is an invasive procedure. Non-invasive urine biomarkers are an active area of investigation to support LN diagnosis and management.

**Objective:**

To investigate the role of urinary galectin-3 binding protein (u-Gal-3BP) as a candidate biomarker of renal disease in biopsy proven LN.

**Patients and methods:**

Levels of u-Gal-3BP were investigated in a cross-sectional fashion by ELISA in 270 subjects: 86 LN patients, 63 active SLE patients with no kidney involvement, 73 SLE patients with inactive disease and 48 age and sex-matched population-based controls (PBC). Moreover, urine samples were analysed separately by ELISA for additional markers of kidney pathology: neutrophil gelatinase-associated lipocalin (NGAL), osteopontin (OPN), kidney injury molecule-1 (KIM-1) and galectin-3 (Gal-3). The concentrations of all studied molecules were normalized to urine creatinine levels. In 10 patients, post-treatment levels of the biomarkers were measured.

**Results:**

Normalized u-Gal-3BP levels were higher in LN patients compared to the other groups (*p < .0001*). Comparing different LN classes, u-Gal-3BP levels were higher among patients with proliferative (class III/IV) and membranous (class V) as compared to mesangial (class II) forms (*p = .04*). In proliferative forms, u-Gal-3BP levels correlated with the activity index in renal biopsies (r = 0.42, *p = .004*). Moreover, in a subset of 10 patients with repeated kidney biopsy and urine sampling before and after induction treatment, a significant decrease of u-Gal-3BP was observed (*p = .03*)*.* Among the other markers, KIM-1 was also able to discriminate LN from the other groups, while NGAL, OPN and Gal-3 could not in this cohort.

**Conclusion:**

Given its ability to discriminate LN patients from active non-renal and inactive SLE patients, the observed correlation with the activity index in renal biopsies, and its levels declining following treatment, u-Gal-3BP shows promise as a non-invasive urinary biomarker to help detecting and to monitor renal involvement in SLE patients and should be validated in larger cohorts.

## Introduction

Lupus nephritis (LN) is a highly prevalent and serious clinical manifestation among patients with systemic lupus erythematosus (SLE). It affects up to 60% of patients, depending on the examined cohort.^1^ Although SLE management has improved, LN still represents a difficult to treat manifestation, with renal flares occurring in about half of the patients and development of end stage renal disease (ESRD) in 5%–20% of patients.^1,2^

Lupus nephritis directly contributes to SLE-related mortality, both in the early and later disease phase.^3^ Indeed, accelerated atherosclerosis, an important factor contributing to premature mortality in SLE, is a feature essentially confined to LN patients as compared to non-nephritis subgroups and the general population.^4^

Lupus nephritis diagnosis relies on kidney biopsy, which is instrumental for histological characterization,^5^ and treatment decisions. However, the biopsy procedure is invasive, often associated with discomfort for the patient, and sometimes with bleeding complications.^6^ Although repeated biopsies have been shown to be of value, their utility still remains controversial for verifying treatment effects, monitoring disease and predicting outcomes in clinical practice.^7^

In this context, it is highly desirable to identify new non-invasive biomarkers that may reflect the type of kidney involvement, reflect the degree of histological activity and damage, predict LN flares and be useful for assessing treatment response. In this respect, urinary biomarkers are of high relevance since they can serve as liquid biopsy and reveal pathogenic events taking place in the kidney.

Some markers already discovered in various types of renal diseases, have been explored also in the context of LN.^8^

We investigated urinary Galectin-3 binding protein (u-Gal-3BP) as a novel candidate biomarker for disease activity in renal lupus, here studied in a real life SLE cohort.

Gal-3BP is an interferon-inducible secreted scavenger protein belonging to the lectin family.^9–11^ In SLE, previous studies have demonstrated high expression of Gal-3BP in blood,^12^ both in systemic and cutaneous forms of lupus,^13^ and the protein was found in circulating microvescicles, as well as in microvesicles in the context of immune deposits in the kidney.^10,14,15^

Considering the relevance of interferon in SLE, and the fact that Gal-3BP is encoded by an interferon inducible gene,^16^ we hypothesized that Gal-3BP could be found as a soluble protein in urine samples of active LN patients and may be used as a marker of kidney involvement and inflammatory activity.

Furthermore, we studied u-Gal-3BP in comparison to Kidney injury molecule-1 (KIM-1), neutrophil gelatinase associated lipocalin (NGAL), osteopontin (OPN) and Galectin-3 (Gal-3), which have been previously explored in SLE-associated renal pathology.^17–20^

## Methods

A total of 222 SLE patients from the Karolinska SLE and nephritis cohorts were included in the study. All patients fulfilled at least four of the 1982 American College of Rheumatology (ACR) and/or the SLICC (Systemic Lupus International Collaborating Clinics) criteria for SLE.^21,22^ The study cohort consisted of urine samples from 86 biopsy-proven LN patients with samples taken at renal biopsy (LN). As controls, samples from 63 SLE patients with active disease but no history of renal involvement (active non-renal SLE, ANR-SLE) and 73 patients with inactive disease and no previous history of LN (inactive non-renal SLE, INR-SLE) were included. In 10 LN patients, additional follow-up samples (n = 10) were obtained at a repeated renal biopsy after immunosuppressive therapy. In each group, disease activity was measured by the SLE Disease Activity Index 2000 (SLEDAI-2K)^23^ at the time of urine sampling. Inactive disease was defined as a SLEDAI-2K 0–2 and active disease as a SLEDAI-2K score of 4 or above with no signs of ongoing renal involvement for the ANR-SLE and with renal involvement for the LN group.

In addition, urine samples from 48 population-based controls (PBC), matched for age and gender to the LN cohort were used.

Demographic and clinical information was collected from the cohort databases and the electronic medical records. Ethical permission was obtained from the Regional Ethics Review Board in Stockholm, and informed consent was obtained from all study subjects. The study complies with the declaration of Helsinki.

### Laboratory variables

Serum creatinine was analysed according to clinical routine at the Karolinska University Hospital Clinical Chemistry Department and expressed as micromoles/liter (μmol/L). Renal function (estimated glomerular filtration rate, eGFR) was calculated using the Chronic Kidney Disease Epidemiology Collaboration equation (CKD-EPI).^24^

Complement levels were determined on a modular analyser (Roche), with normal ranges of 0.67–1.29 g/L for C3 and 0.13–0.32 g/L for C4.

In the LN group, a subset of patients was sampled before establishing this method. Such samples were analysed by nephelometry array (Beckman Coulter). The normal level using this method was 0.5–1.2 g/L for C3 and of 0.1–0.4 g/L for C4.

Anti-dsDNA antibodies were routinely analysed over the years using different methods at the Department of Clinical Immunology at the Karolinska University Hospital.

For statistical purposes, and considering the changes in laboratory methods, C3, C4 and anti-dsDNA were considered as categorical variables in this study, based on their being outside or within reference ranges.

### Analysis of urine biomarkers

Urine galectin-3 binding protein levels were determined using an ELISA kit from MilliporeSigma (Cat # SPRCUS866, St Louis, MO, USA) according to the manufacturer’s instructions. The assay is specific for human Gal-3BP, has been validated for urine testing and can detect the analyte at a minimum detectable concentration of 0.08 ng/mL. Furthermore, we investigated other markers of renal pathology including, NGAL, OPN, KIM-1 and Gal-3 using commercial sandwich ELISAs (DY1757, DY1433, DY1750B and DY1154, R&D Systems, Minneapolis, MN, USA). Urine-albumin and urine-creatinine were determined on a Mindray BS-380 (Shenzhen Mindray Bio-medical Electronics, Shenzhen, China) using reagents from Abbott Laboratories (Abbott Park, IL, USA).

Urine-albumin/creatinine ratio (u-ACR) was calculated from the original values of the ratio determinants and expressed as mg/mmol. All biomarkers were analysed separately and values were normalized as concentration/u-creatinine levels.

All urine investigations were performed according to clinical routine at the Department of Clinical Chemistry at Uppsala University Hospital.

### Histopathological evaluation

Kidney biopsies from the SLE patients were obtained by ultrasound-guided biopsy. Histopathological evaluation was performed at the Pathology Unit of the Karolinska University Hospital. The biopsy specimens were classified according to the International Society of Nephrology/Renal Pathology Society (ISN/RPS) classification.^5^ Activity and chronicity index^25^ were also evaluated.

### Statistical analysis

Continuous variables are described as median and interquartile range (IQR) or 5th–95th percentile interval (in figures) in compliance with normality test. Categorical variables are described as numbers and/or percentages. Differences between groups (unrelated samples) were tested through the Mann–Whitney U-test. Comparisons across several groups were made using the Kruskal–Wallis test. To compare between timepoints, Wilcoxon signed rank test was applied. Correlation analysis was conducted by Spearman’s test. Simple linear regression and ROC curve analysis were applied as appropriate to further explore relations between variables and performance of the investigated biomarker. *p* values of less than .05 were deemed as significant.

All urine biomarkers calculations included all values within the limits of detection, while excluding values under and above this range. In all groups, and for all biomarkers, more than 70% of values fell within the quantification range.

## Results

### Characteristics of SLE patients

Demographic and clinical characteristics of the SLE patients and controls are detailed in Table 1. The two active SLE groups (LN and ANR-SLE) were comparable concerning age and sex distribution. The inactive patients (INR-SLE) were older (*p < .0001*) and had longer disease duration (*p < .0001*) compared to the other two SLE groups (Table 1). Of note, in 37/86 (43%) patients, LN occurred at the onset of SLE. Renal function, expressed by serum creatinine levels and eGFR values, did not differ across the study groups (*p* = .18 and *p* = .09, respectively).

**Table 1.** Clinical characteristics at inclusion in lupus nephritis, SLE control groups and population-based controls.

	Lupus nephritis (n = 86)	Active non-renal SLE (n = 63)	Inactive non-renal SLE (n = 73)	Population-based controls (n = 48)	Comparison across groups^a^ (*p*)
Age (years)	35.5 (26.0-43-0)	37.0 (28.0–46.0)	51.0 (30.5–59.5)	34.5 (28.0–46.7)	*<0.0001*
Female, (%)	89.5	90.5	89.0	91.7	0.97
Ethnicity, (%)					
Caucasian	76.7	93.6	87.7	95.8	*0.05*
Asian	10.5	1.6	5.5	0	
African	8.1	4.8	2.7	2.1	
Hispanic	4.6	0	4.1	2.1	
Disease duration (years)	2.0 (0.0–8.0)	5.0 (0.0–8.0)	8.0 (4.0–17.5)	—	*<0.0001*
SLEDAI-2K	12.0 (8.0–17.7)	6.0 (4.0–8.0)	1.0 (0.0–2.0)	NA	*<0.0001*
S-creatinine, (⌈mol/L)	68.0 (57.5–86.0)	66.0 (58.0–73.0)	67.0 (58.0–76.5)	62.5 (56.2–70.7)	0.18
GFR, (mL/min/1.73 m^2^)	101.8 (77.5–122.8)	103.2 (89.2–121.5)	95.0 (78.8–109.1)	NA	0.09
U-ACR (mg/mmol)	46.0 (19.1–76.6)	2.8 (1.6–3.7)	1.4 (0.9–2.4)	NA	*<0.0001*
Anti-ds DNA positive, (%)	71.4^b^	63.0^b^	40.5^b^	NA	*0.004*
C3 low, (%)	64.9^c^	25.0^c^	45.0^c^	NA	*<0.0001*
C4 low, (%)	75.4^d^	7.2^d^	21.7^d^	NA	*<0.0001*
Ongoing treatment					
Prednisolone, (%), mg daily	73.2 10.0 (5.0–10.0)	10.0 (5.0–15.0)	39.7 6.2 (4.4–10.0)	NA	*<0.0001* *0.01*
Antimalarials, (%)	45.3	60.3	47.9	NA	0.17
DMARD any, (%)	44.2	50.7	24.6	NA	*0.004*
Azathioprine, (%)	8.1	17.5	15.1	NA	0.20
Methotrexate, (%)	5.8	10.0	6.8	NA	0.08
Cyclophosphamide, (%)	2.3	7.9	2.7	NA	0.18
Mycophenolate mofetil, (%)	19.8	9.5	2.7	NA	*0.003*
Cyclosporine A, (%)	1.2	0	0	NA	—
BCDT, (%)	7^e^	0	0	NA	—

Continuous variables are expressed as M (IQR): median (interquartile range); categorical variables are expressed as percentages.

NA: not assessable; SLEDAI-2K: Systemic Lupus Erythematosus Disease Activity Index-2000; GFR: glomerular filtration rate, estimated by the CKD-EPI formula (add ref number); Anti-dsDNA: anti-double strand DNA antibodies; C3, C4: complement fractions 3 and 4; DMARD: Disease Modifying Anti-Rheumatic Drugs; BCDT: B cell depletion therapy.

Italic characters refer to statistically significant *p* values.

^a^comparison across groups: Kruskal–Wallis test, chi-square test.

^b^data available for 77, 46 and 42 patients

^c^data available in 57, 60 and 69 patients.

^d^data available in 57, 60 and 69 patients.

^e^Rituximab in all but one case (ofatumumab). One case RTX-MMF, one case RTX-CYC.

The LN patients were, as expected, anti-dsDNA positive and showed complement activation in higher proportion than the other two SLE groups (*p = .004* and *p < .0001*). The treatments ongoing at recruitment in each SLE group are presented in Table 1.

### Urine galectin-3 binding protein distinguishes LN from the other SLE groups and PBCs

**Figure 1.**
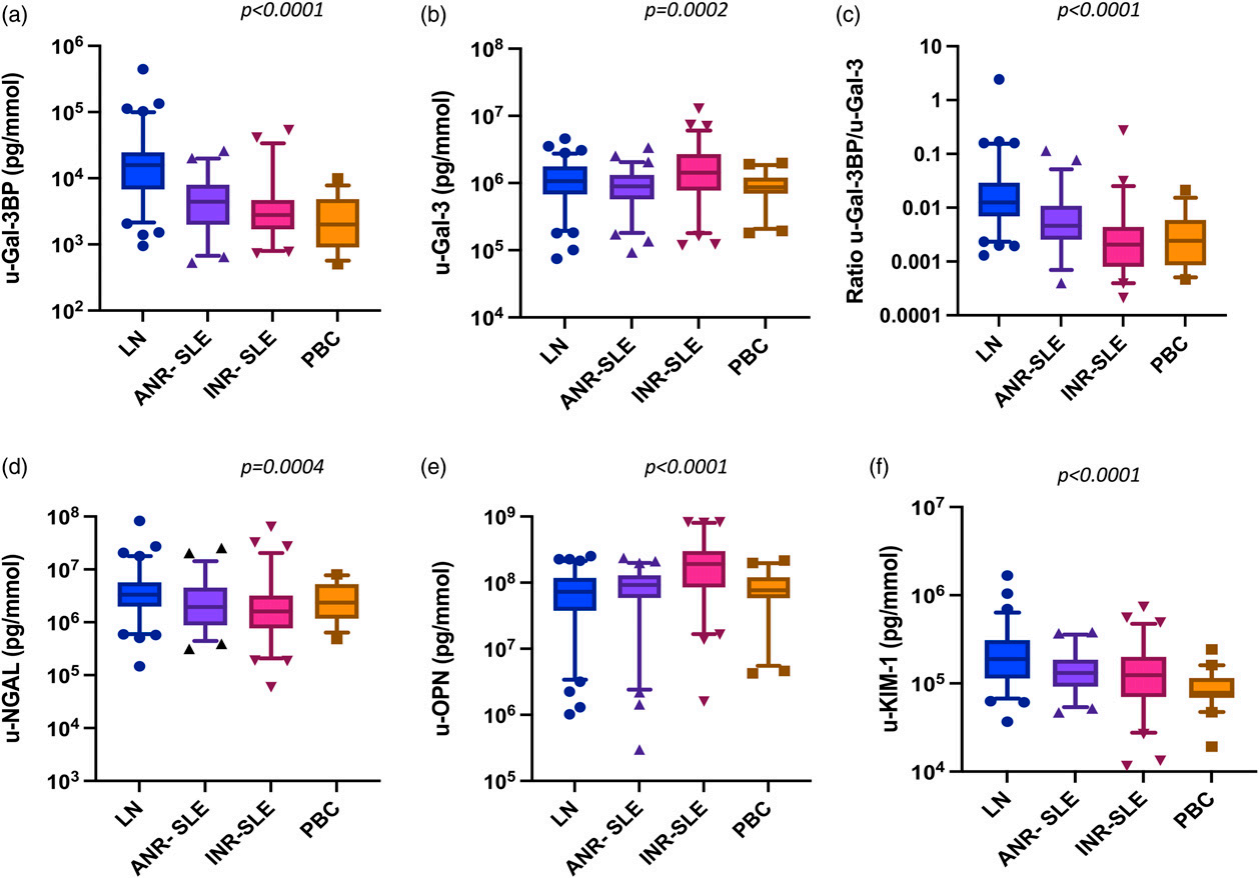
Urine biomarker levels adjusted for urine creatinine concentration in SLE patients and population-based controls. A–F: each panel shows the median and 9th–95th percentile values of each tested urinary biomarker in the study patients divided into lupus nephritis (LN), active non-renal SLE (ANR-SLE), inactive SLE (INR-SLE) and population-based controls (PBC). Values are normalized to urine-creatinine concentration and expressed as pg/mmol. Y axes are in logarithmic scale. *p* values show significance in the Kruskal–Wallis test for comparison across groups.

Unless otherwise specified, levels of the biomarkers mentioned below were normalized for urine creatinine concentrations. Levels are reported in Figure 1 and Supplementary Table 1.

Lupus nephritis patients showed higher levels of u-Gal-3BP compared to the other SLE groups and controls (*p < .0001*, Figure 1(a)). Such difference was evident even when the levels of u-Gal-3BP were compared between LN patients and each of the other groups at a time (*p < .0001* for all). Moreover, the ability of u-Gal-3BP to distinguish LN from ANR disease was further explored by ROC analysis, showing an AUC = 0.82 (*p < .0001*), (Supplementary Figure 1). No differences were observed between ANR-SLE and INR-SLE (*p* = .07), which both showed higher u-Gal-3BP levels than the PBC (*p = 0.001* and *0.04*, respectively). Urine galectin-3 levels were different across groups (*p = .0002*, Figure 1(b)); however, the levels in LN patients could not discriminate this group from ANR-SLE (*p* = .09). As for u-Gal-3BP, the ratio between the protein and its binding partner Gal-3 (u-Gal-3BP/u-Gal-3), also differed across the groups (*p < .0001*, Figure 1(c)), with LN patients showing higher ratios than ANR-SLE, INR-SLE patients and PBC (*p < .0001* for each comparison). To evaluate whether u-Gal-3BP levels could simply be a passive reflection of ongoing renal protein leakage, we examined the relationship between u-Gal-3BP and u-ACR by linear regression. This analysis showed non-significant results with R^2 = 0.03 ad *p* = .13. Similarly to u-Gal-3BP, u-NGAL levels differed across the groups (*p = .0004*, Figure 1(d)). However, while u-NGAL was higher in LN compared to ANR-SLE patients (*p = .008*) and INR-SLE (*p < .0001*), this urine biomarker showed similar levels in LN patients and PBC (*p* = .08). Moreover, u-NGAL levels were similar between ANR-SLE and INR-SLE (*p* = .26) and both groups did not differ from PBC (*p* = .45 and 0.08, respectively, data not shown).

U-OPN levels, which differed across groups (*p < .0001*, Figure 1(e)), were highest in INR-SLE. This group showed higher levels compared to LN, ANR-SLE patients and PBC (*p < .0001* for each comparison). No difference in u-OPN emerged comparing LN and ANR-SLE patients (*p* = .16).

U-KIM-1 levels (Figure 1(f)) differed across the groups (*p < .0001*), with higher values in LN compared to ANR-SLE (*p = .004*), INR-SLE (*p = .0005*) and PBC (*p < .0001*).

As expected, significant differences were seen in u-ACR across the groups (*p < .0001*) with increased levels in LN compared to the other subgroups (*p < .0001* for each comparison), (Supplementary Table 1).

Analysis of biomarker levels not normalized to u-creatinine is reported in Supplementary Figure 2: briefly, u-Gal-3BP concentrations showed similar behaviour compared to u-creatinine adjusted levels (Supplementary Figure 2A), with LN driving the difference against each group (*p < .0001* for all).

### Histopathological description of the LN biopsies

The partition of LN patients according to histopathological findings is illustrated in Supplementary Figure 3. Briefly, there were 47 patients with proliferative forms (PN), and 26 patients with membranous forms (MN). Twelve patients with inactive LN not requiring increased immunosuppressive treatment (10 with ISN/RPS class II, one class III C, one class IV-S(C)) were grouped as mesangial LN (MES). One patient was classified as “other” since the histological picture was consistent with glomerulosclerosis with no signs of inflammatory activity. Overall, the median (IQR) activity index was 3 (1–5), with a maximum score in the cohort of LN of 13. The median chronicity index was 0 (0–2), with a maximum observed score of 5 in this cohort. Within the PN subgroup, median (IQR) activity index was of 4 (3–6), while the chronicity index showed median (IQR) values of 0 (0–5).

### Urine galectin-3 binding protein helps distinguish proliferative LN from other histopathological subtypes

**Figure 2.**
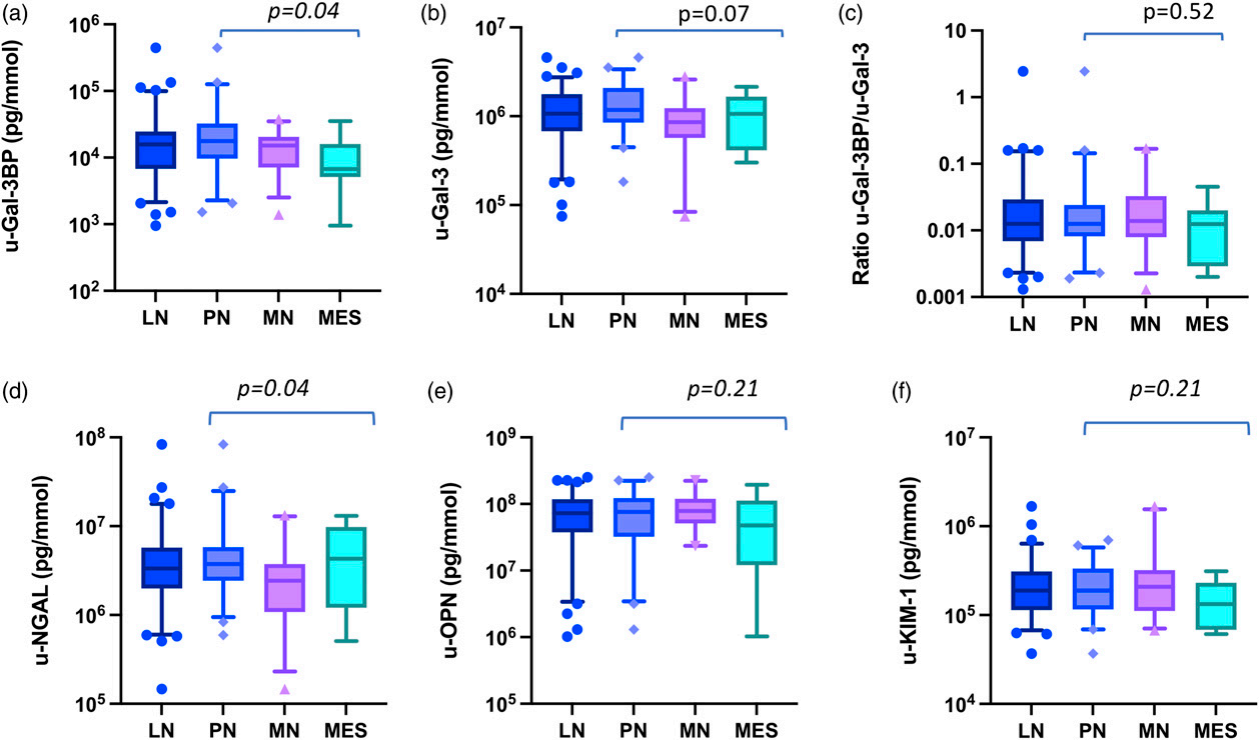
Urine biomarker levels adjusted for urine creatinine concentration in lupus nephritis subtypes. A–F: each panel shows the median and 9–95th percentile values of each tested urinary biomarker in the lupus nephritis (LN) patient group and in the LN subtypes of proliferative nephritis (PN), membranous nephritis (MN) and mesangial nephritis (MES). Biomarker urinary values are adjusted for urine-creatinine concentration and expressed as pg/mmol. Y axes are in logarithmic scale. *p* values show significance in the Kruskal–Wallis test for comparison across groups and refer to comparisons across the LN subtypes.

We next investigated how the assessed biomarkers relate to the major subtypes of LN (Figure 2).

Urine galectin-3 binding protein levels were different across LN subtypes (*p = .04*). They were higher in PN as compared to MES (*p = .03*), however, no significant difference was detected between PN and MN (*p* = .11) or between MN and MES (*p* = .12, Figure 2(a) and Supplementary Table 2). U-Gal-3 levels did not show differences across groups (*p* = .07, Figure 2(b)), although PN patients showed higher levels than MN (*p = .03*). The ratio u-Gal-3BP/u-Gal-3 did not differ across groups, nor did the levels of u-OPN and u-KIM-1 (Figure 2(c), (e) and (f)).

When compared across groups, u-NGAL showed dif discriminating PN against MN (*p = .01*), comparable levels were found between PN and MES (*p* = .88).

The values of each biomarker not adjusted for urine creatinine in the LN subtypes, are depicted in Supplementary Figure 4. No differences were observed for u-Gal-3BP, u-Gal-3, the ratio u-Gal-3BP/u-Gal-3 and OPN (panel A, B, C and E).

Differences (Supplementary Figure 4 (D) and (F)) were detected in the levels of u-NGAL (*p = .04*) and KIM-1(*p = .03*). The former was higher in PN compared to MN (*p = .01*), but comparable between PN and MES (*p* = .26). The latter was mostly expressed in MN compared to MES (*p = .01*), while PN levels were not different from those of MN (*p* = .07) nor MES (*p* = .11).

### Urine galectin-3 binding protein is associated with the degree of activity at renal biopsy

We next explored how the levels of adjusted urinary biomarkers correlated with histological activity (activity index), in patients with PN.

Urine galectin-3 binding protein showed a moderate correlation (r = 0.42, 95% CI 0.13–0.64, *p = .004*) with the activity index in PN patients (n = 47). No associations were detected for the levels of Gal-3, NGAL, OPN, KIM-1 and u-ACR. For PN not on DMARDs (corticosteroid allowed, n = 31), the association between activity index and u-Gal-3BP was stronger (r = 0.47, 95% CI 0.19–0.72, *p = .009*). There was no correlation in PN patients regarding any urine biomarker and chronicity score (data not shown).

### Urine galectin-3 binding protein levels are influenced by ongoing corticosteroids but not immunosuppressants

**Figure 3.**
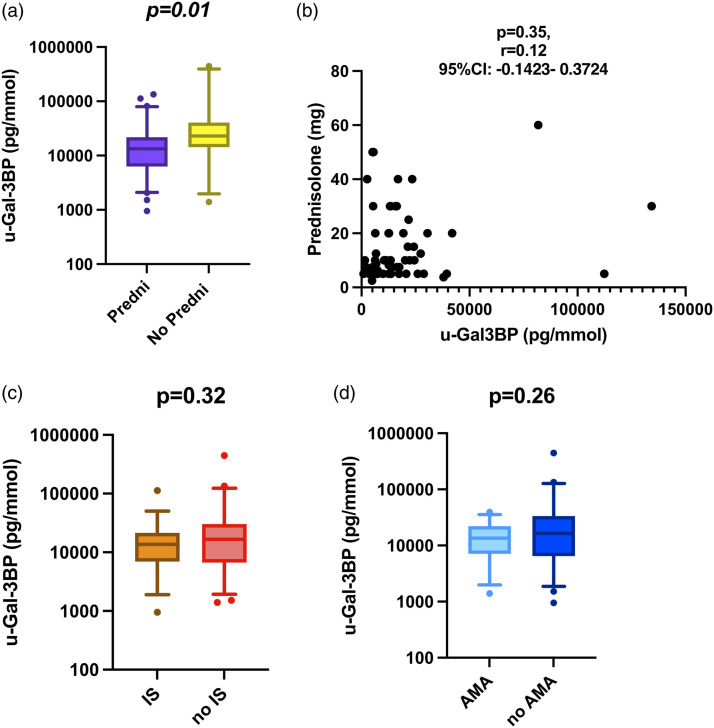
Galectin-3 binding protein levels in relation to ongoing treatments at urine sampling in lupus nephritis. Panel A–D: (a): in the whole LN group, levels of u-Gal-3BP were compared in patients receiving oral corticosteroids (Predni) with respect to those not receiving corticosteroids (No Predni); (b) for those on oral corticosteroids, Spearman’s test was run to ascertain any correlation between levels of u-Gal-3BP and dose of ongoing prednisolone; similarly (c), levels of u-Gal-3BP were compared in patients undergoing immunosuppressive treatment (IS) or not at the time of sampling; the same comparison was performed for patients with LN on antimalarial (AMA) treatment with respect to those not on AMA treatment.

In the next step, we analysed the impact of ongoing treatments on the levels of the urinary biomarkers (Figure 3). Patients who were under oral corticosteroids at the time of kidney biopsy, showed lower median levels of adjusted u-Gal3BP compared to those who did not (Figure 3(a)). Despite this association, no correlation was observed between u-Gal-3BP levels and the daily corticosteroid dose (Figure 3(b)). Adjusted u-Gal-3, -NGAL, -OPN, and -KIM-1 were not influenced by oral corticosteroid treatment (data not shown). Being on immunosuppressants (IS) or antimalarial (AMA) therapy was not associated with significant differences in u-Gal-3BP (Figure 3(c) and (d)), Gal-3, NGAL, OPN or KIM-1 levels (data not shown).

### Corticosteroids and antimalarials influence u-Gal-3BP expression in PN

**Figure 4.**
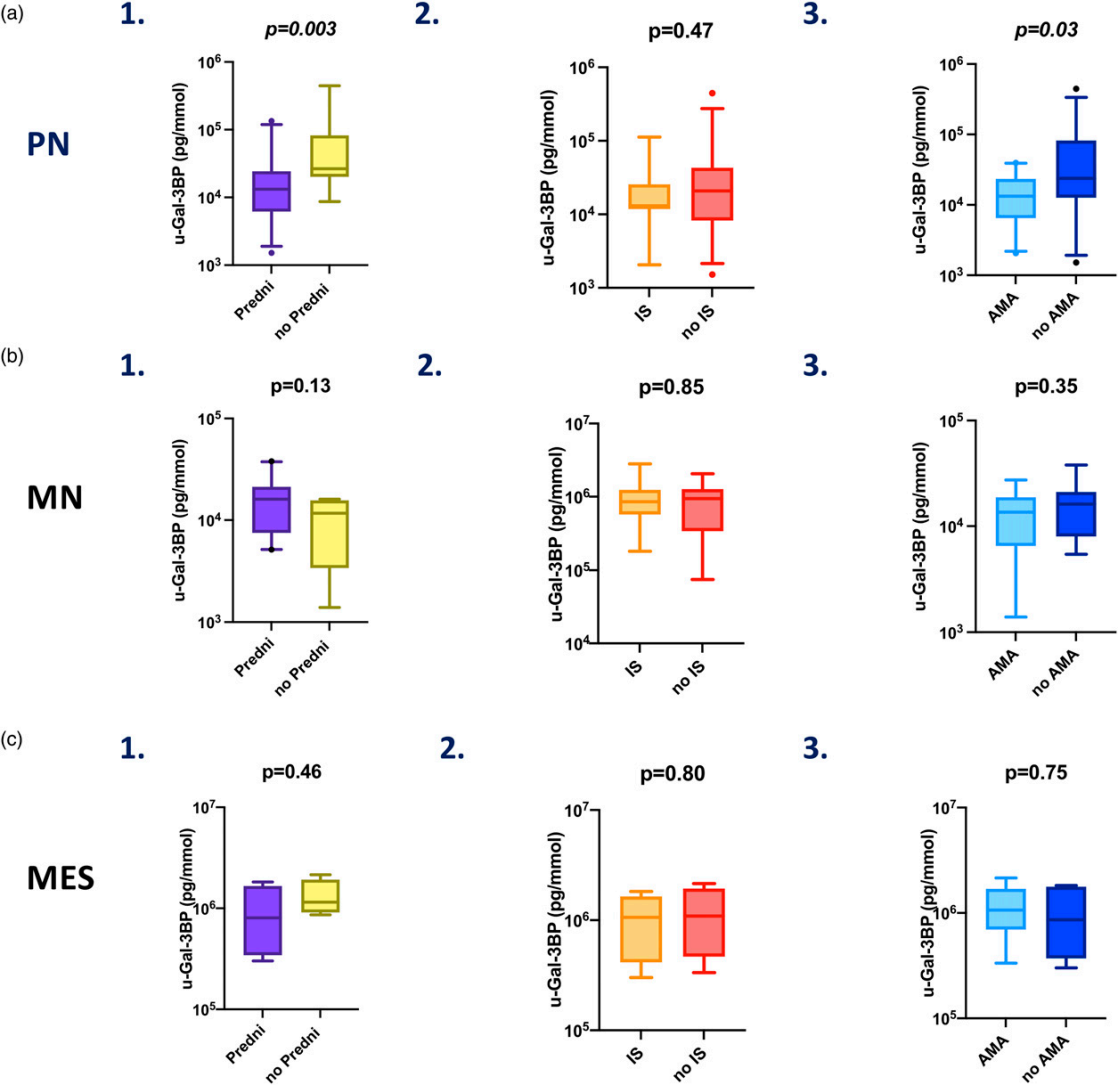
u-Galectin-3 binding protein adjusted levels in relation to ongoing treatments at urine sampling in LN subtypes. Panel (a) in PN, levels of u-Gal-3BP were compared in patients on active versus non-active treatment at the time of sampling with (1) corticosteroids (Predni), (2) immunosuppressants (IS) and (3) antimalarials (AMA). Panel (b) in MN, similarly, u-Gal-3BP levels were compared in patients on active versus non-active treatment with (1) corticosteroids (Predni), (2) immunosuppressants (IS) or (3) antimalarials (AMA). Panel (c) in MES, similar comparison was performed for u-Gal-3BP levels in patients on active versus non-active treatment with (1) corticosteroids (Predni), (2) immunosuppressants (IS) and (3) antimalarials (AMA).

We next explored whether the difference in u-Gal-3BP levels was present independently or not from the subtype of LN determined by histological examination. The difference (Figure 4) was mainly driven by the PN group, in which corticosteroids were still associated with lower levels of u-Gal-3BP *(p = .003)* while no difference was observed in MN (*p* = .13) or MES (*p* = .46). Ongoing DMARDs treatment was not associated with lower levels of adjusted u-Gal-3BP in either PN, MN nor MES (Figure 4).

Antimalarial treatment did not influence adjusted levels of u-Gal-3BP in MN and MES (Figure 4, B3, C3). However, when only PN was considered (Figure 4, A3), the levels of u-Gal-3BP were lower in patients receiving antimalarial treatment *(p = .03).*

### Urine galectin-3 binding protein levels decrease upon LN induction treatment

In 10 LN patients, samples were available both at active renal flare, when induction treatment was started, and at a follow-up biopsy, performed after a median (IQR) time of 7.5 (6–13) months after the first biopsy. We found a significant decrease in adjusted median u-Gal-3BP levels in these patients, (*p = .03*, Supplementary Table 3). The other tested biomarkers did not show a statistically significant decline in their urinary levels, with the exception of KIM-1 which decreased significantly (*p = .006,* data not shown).

## Discussion

In this study, higher levels of u-Gal-3BP were detected in patients with LN as compared to other SLE patients and PBCs. This new data is in agreement with a recent report about u-Gal-3BP, where urinary levels of this marker were found to be higher cross-sectionally in LN patients compared to healthy controls and CKD patients.^26^ Importantly, our study included active non-renal SLE as a comparator. Furthermore, longitudinal data was obtained in this study, although only for a small subset of patients. More specifically, among LN patients, u-Gal-3BP levels were highest in the PN and MN subtypes, and they correlated with the degree of histological activity, suggesting pathogenic implications for u-Gal-3BP. There was also a negative association with corticosteroid use and clear reduction of u-Gal-3BP following induction therapy. Taken together, our results suggest that u-Gal-3BP is a potential non-invasive biomarker suitable for surveillance and monitoring of renal activity in LN. In previous studies in SLE, serum and plasma levels of Gal-3BP correlated with disease activity and with activation of interferon-related genes.^12^ In a recent study, the release of DNA-segments and Gal-3BP containing microvesicles was triggered upon stimulation of peripheral blood mononuclear cells of SLE patients by IFN-alpha, as well as via TLR-7 and TLR-9 agonists,^15^ which reinforces the concept of Gal-3BP as an interferon inducible protein directly involved in pathogenic events in SLE. The protein has also been studied as a component of circulating microvesicles and was found to be present in immune deposits in kidney biopsies of LN patients, where it co-localizes with immunoglobulins.^14,27^ In a recent study, high serum levels of Gal-3BP were found to be a major predictor of incident venous thromboembolism in a Swedish longitudinal cohort of 162 SLE patients.^28^

The source of the protein however remains unclear. Although previous studies suggest that the protein might be transported into kidney tissue via microvesicles,^14^ it is also possible that it is secreted in kidney resident or infiltrating immune cells, driven by type I IFN and the activation of IFN-inducible genes. In this case, u-Gal-3BP increases would be a direct reflection of active renal inflammation.

Studies focussing on Gal-3BP in renal pathology are limited, while more is known about its ligand Gal-3.^19^ To our knowledge, no data is available about its expression and possible role as biomarker in common causes of chronic renal pathology, such as hypertension-related glomerulosclerosis or diabetic nephropathy. Of note, in the study by Ding H. et al., the CKD patient group showed lower levels of this marker in the urine compared to LN patients.^26^ Some additional data is available concerning toxic renal damage, where the protein has been found in exosomes in urine samples.^29^ Moreover, increased plasma levels of the protein have been described in acute Hantavirus infection, which typically causes renal failure.^30^

Apart from the biological implications of Gal-3BP presence in urine samples of SLE patients, it is interesting to consider its possible performance as a biomarker of kidney involvement. Currently, the assessment of renal activity relies on clinical parameters such as grade of proteinuria and cellular casts, neither of which is specific or sensitive for SLE-related kidney pathology. Although responsive to change and predictive of long-term clinical outcomes,^31,32^ proteinuria does not mirror the type of nephritis, nor can it discriminate active disease from residual chronic damage. Serological activity markers such as increases in anti-dsDNA antibodies and complement consumption are generally considered associated with renal activity, but do not prove to be reliable markers of activity in LN.^33^

Several molecules, as well as cells and nucleic acid (e.g. miRNAs) expression have been investigated over the years as LN biomarkers,^8^ some with promising results, although their clinical application remains limited. In a recent large screening of candidate urinary biomarkers in active LN, E-selectin, VCAM-1, BFL-1 and hemopexin were increased among Caucasian patients;^34^ however, the samples were not taken at time of renal biopsy and data on ongoing renal histopathology were not available. In our study, urine samples were taken at the time of renal biopsies, thus, we can in a more reliable way associate them with ongoing processes in the renal tissue.

The other markers explored here were not able to allow distinction between LN and other disease categories or controls. Osteopontin for instance, although increased in SLE patients in other studies and associated with disease activity and specific clinical manifestations^35,36^ was in our study mostly expressed in inactive SLE patients, which was rather unexpected, considering its role as an inflammation marker.^37^ A recent small study found OPN expressed at higher levels in urine than in the serum of SLE patient, with the serum protein being a possible marker of LN presence, but unable to correlate with the phase of renal activity.^18^

The highest levels of u-KIM-1 were seen among LN patients as compared to other SLE subsets and controls. Being a marker of renal and tubular injury, this was not unexpected. NGAL, another marker of renal injury, although able to discriminate between LN and active and non-active SLE, was not able to differentiate LN from PBC in our study. Galectin-3, one of the ligands of G3BP, did not discriminate ANR-SLE from LN and the ratio of G3BP/Gal-3, employed to capture differential variations in the two molecules, did not outperform G3BP alone in our sample.

Exploring the effects of baseline treatments and therapeutic interventions after kidney biopsy, we could see that ongoing corticosteroids reduce the expression of u-Gal-3BP, which could be explained by the suppression of interferon inducible genes possibly determined by these drugs.^38^ On the other hand, antimalarials, which are thought to influence interferon-related mechanisms,^39^ did not show a strong influence on Gal-3BP urinary expression, apart from PN where antimalarial use was associated with lower levels of U-Gal-3BP. Similarly, immunosuppressive treatments at baseline were not associated with lower levels of u-Gal-3BP. Interestingly, we observed decreased levels after induction treatment, which indicates that u-Gal-3BP may be used to monitor treatment effects, though larger longitudinal studies are needed, also to assess u-Gal-3BP performance in flare prediction.

This is a cross-sectional real-world study and thereby also includes patients already on immunosuppressive treatment, which may have influenced the results. Methods for anti-dsDNA and complement components have varied over time, thus making comparisons with conventional serum biomarkers difficult. Moreover, plasma levels of G3BP were not measured for this study, with previous literature providing the background for their utility.^12^ On the other hand, this study focused on urinary biomarkers, considering the advantageous properties of using urine samples as a non-invasive, easily collectable source of information in SLE and LN patients. The strengths of the study are the sample size, the fact that samples were obtained at renal biopsy thus reflecting the ongoing renal activity, and the availability of ANR-SLE as comparator.

In conclusion, we demonstrate that u-Gal-3BP is a good marker of renal disease in SLE. Additionally, u-Gal-3BP reports on the type of renal inflammation and level of activity, both by showing elevated levels during active disease, and by declining following immunosuppressive treatment. Since urine samples are non-invasive and easily accessible, our findings suggest that u-Gal-3BP is an interesting marker to further explore in larger and longitudinal studies.

## Supplemental Material

Supplemental Material - Urine Galectin-3 binding protein reflects nephritis activity in systemic lupus erythematosus Click here for additional data file. Supplemental Material for UUrine Galectin-3 binding protein reflects nephritis activity in systemic lupus erythematosus by Francesca Faustini, Helena Idborg, Enrico Fuzzi, Anders Larsson, Wen-Rong Lie, Sven Pötzsch, Shinji L Okitsu, Elisabet Svenungsson and Iva Gunnarsson in Lupus
