# Supplementary material for: Urine Galectin-3 binding protein reflects nephritis activity in systemic lupus erythematosus
Source: Lupus. 2022 Dec 12;32(2):252–62. doi: 10.1177/09612033221145534 (PMC9939930; doi:10.1177/09612033221145534)
Supplement: Supplemental Material - Urine Galectin-3 binding protein reflects nephritis activity in systemic lupus erythematosus [file sj-pdf-1-lup-10.1177_09612033221145534.pdf]

Supplementary Figure 1. Receiver Operating Curve (ROC) for u-Gal-3BP

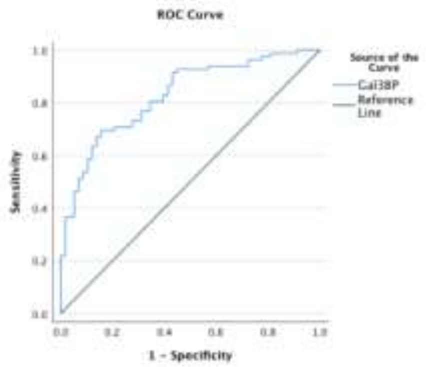

ROC curve of adjusted u-Gal-3BP for predicting active LN against active non renal SLE; AUC=0.82, p<0.0001 95%CI: 0.76-0.89, corresponding to 5501.49 pg/mmol. AUC=area under the curve

Supplementary Figure 2: Urine biomarkers concentration not adjusted for urine-creatinine levels

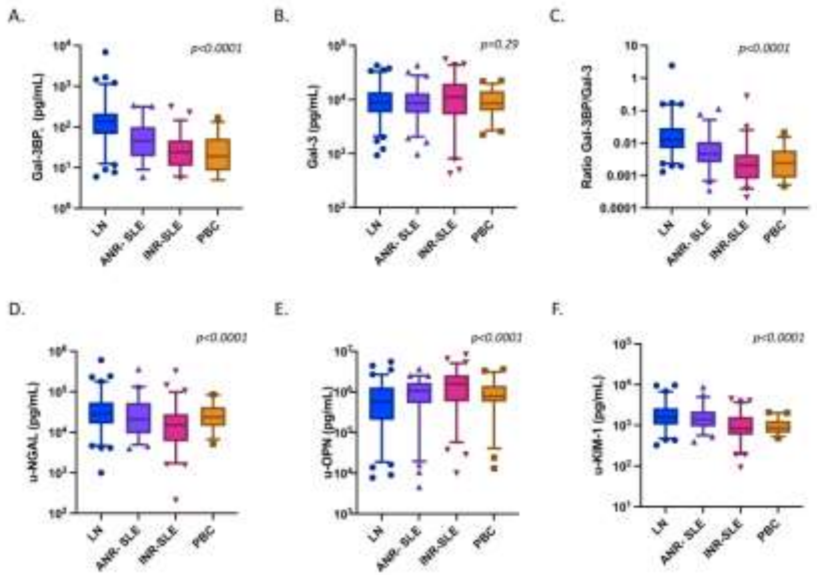

A-F: each panel shows the median and 9-95th percentile values of each tested urinary biomarker in the study patients divided into lupus nephritis (LN), active non renal SLE (ANR-SLE), inactive SLE (INR-SLE) and population-based controls (PBC). Values are not normalized to urine-creatinine concentration and are expressed as pg/mL. Y axes are in logarithmic scale. P values show significance in the Kruskal-Wallis test for comparison across groups.

Supplementary Figure 3: Partition of the lupus nephritis patients according to histopathology findings

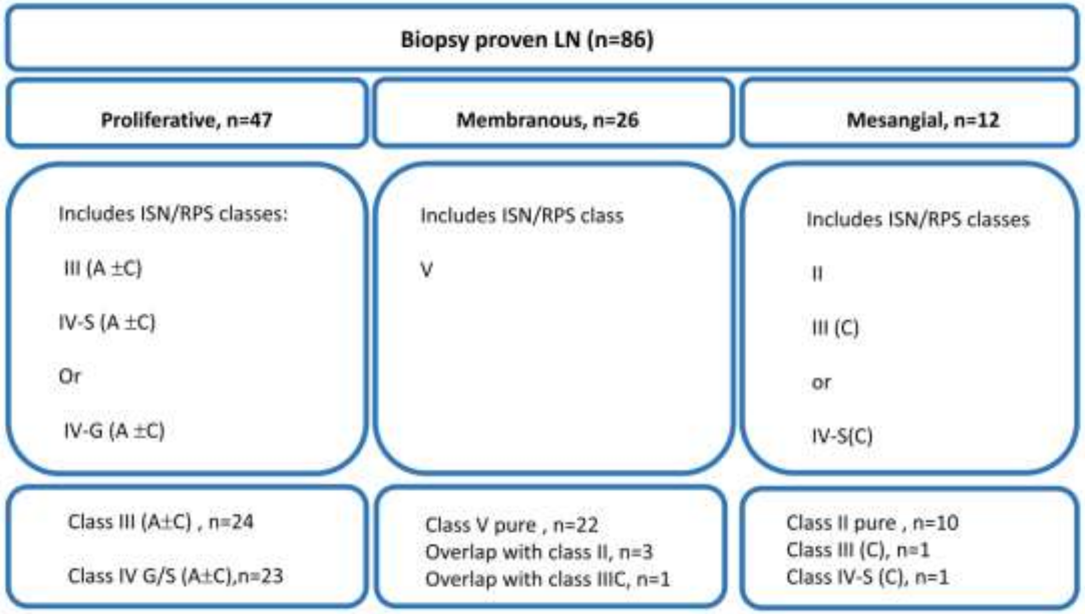

The classes of LN defined according to the ISN/RPS classification system are assigned to the proliferative forms when acute changes are present with or without chronic changes. Class III with only chronic changes or IV with segmental chronic changes have been also assigned to the mesangial group (not requiring increased treatment). In the membranous class pure class V were assigned.

Supplementary Figure 4: Urine biomarkers concentration not adjusted for urine-creatinine levels in lupus nephritis subtypes

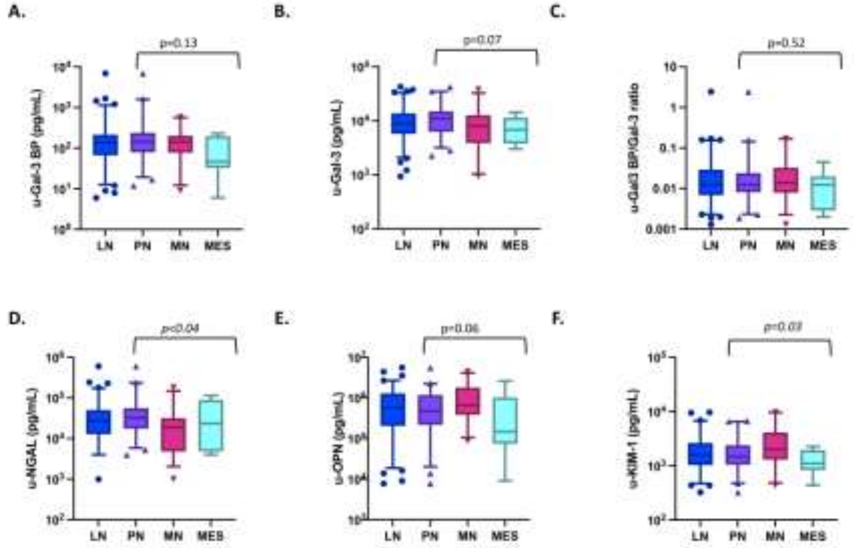

A-F: each panel shows are represented the median and 9-95th percentile values of each tested urinary biomarker in the lupus nephritis (LN) patient group and in the LN subtypes of proliferative nephritis (PN), membranous nephritis (MN) and mesangial nephritis (MES). Values are not adjusted for urine-creatinine concentration and expressed as pg/mL. Y axes are in logarithmic scale. P values show significance in the Kruskal-Wallis test for comparison across groups and refer to comparisons across the LN subtypes.

Supplementary Table 1. Adjusted urine biomarkers in the study subgroups

	LN (N=86)	Active non-renal SLE (N=63)	Inactive SLE (N=73)	Population-based controls (N=48)	Comparison across groups, p-value *
u-GAL3BP (ng/mmol)	n=82<br>15.8<br>(6.8-24.6)	n=58<br>4.4<br>(2.0-9.0)	n=52<br>2.8<br>(1.7-4.7)	n=35<br>2.0<br>(0.9-4.8)	*<0.0001*
u-GAL3 (µg/mmol)	n=85<br>1.1<br>(0.7-1.8)	n=62<br>0.9<br>(0.6-1.3)	n=70<br>1.4<br>(0.8-2.7)	n=48<br>0.9<br>(0.7-1.2)	*0.0002*
u-NGAL (µg/mmol)*	n=80<br>3.3<br>(2.0-5.7)	n=55<br>2.0<br>(0.9-4.5)	n=69<br>1.6<br>(0.8-3.2)	n=35<br>2.4<br>(1.2-5.3)	*0.004*
u-OPN (µg/mmol)	n=84<br>72.98<br>(37.6-118.1)	n=62<br>92.3<br>(58.5-129.7)	n=72<br>190.6<br>(85.1-299.9)	n=47<br>76.5<br>(58.2-120.3)	*<0.0001*
u-KIM-1 (ng/mmol)	n=79<br>188.9	n=55<br>131.4	n=69<br>123.8	n=40<br>78.2	*<0.0001*

	(113.7-309.7)	(92.2-186.1)	(70.3-200.2)	(68.8-115.1)	
u-albumin/creatinine ratio (mg/mmol)	n=86<br>46.97<br>(19.10-76.50)	n=63<br>2.78<br>(1.56-3.75)	n=73<br>1.41<br>(0.93-2.43)	n=48<br>2.33<br>(1.99-2.72)	*<0.0001*
u-Gal3BP/u-Gal3	n=81<br>0.012<br>(0.007-0.029)	n=57<br>0.0046<br>(0.003-0.010)	n=52<br>0.002<br>(0.000-0.004)	n=35<br>0.002<br>(0.000-0.006)	*<0.0001*

Values are reported as M (IQR), median and interquartile range. Calculations were made including results falling in the quantification range as detailed in the method section. All biomarkers values are normalized to urine-creatinine levels, i.e. expressed as weight units per mmol of urine creatinine.

Supplementary Table 2. Adjusted urine biomarkers in all LN patients and in different histopathological subsets

	All LN (N=86)	PN (N=47)	MN (N=26)	MES (N=12)	Other (N=1)
u-Gal-3BP ng/mmol creatinine	n=82<br>15.8<br>(6.8-24.6)	n=46<br>17.7<br>(9.6-32.5)	n=25<br>15.2<br>(7.1-20.5)	n=11<br>6.7<br>(5.1-16.1)	N=1<br>NA
u-Gal-3 µg/mmol creatinine	n=85<br>1.1	n=46<br>1.2	n=26<br>0.8	n=12<br>1.1	NA

	(0.7-1.8)	(0.8-2.1)	(0.6-1.2)	(0.4-1.7)	
u-NGAL<br>*µg/mmol creatinine*	n=80<br>3.3<br>(2.0-5.7)	n=46<br>3.7<br>(2.4-5.8)	n=22<br>2.4<br>(1.1-3.8)	n=11<br>4.3<br>(1.2-9.8)	NA
u-osteopontin<br>*µg/mmol creatinine*	n=84<br>72.98<br>(37.6-118.1)	n=45<br>75.93<br>(32.1-122.8)	n=26<br>78.24<br>(51.2-119.6)	n=12<br>47.17<br>(12.0-111.8)	NA
u-KIM<br>*ng/mmol creatinine*	n=79<br>188.9<br>(113.7-309.7)	n=44<br>189.3<br>(115.7-334.8)	n=23<br>208.2<br>(111.1-321.1)	n=11<br>133.2<br>(68.1-230.9)	NA
u-albumine/creatinine ratio	n=86<br>46.97<br>(19.10-76.50)	n=47<br>52.30<br>(38.16-87.14)	n=26<br>26.00<br>(13.86-67.83)	n=12<br>22.44<br>(9.06-57.61)	NA
U-Gal-3BP/u-Gal-3	n=81<br>0.012<br>(0.007-0.029)	n=45<br>0.012<br>(0.008-0.024)	n=25<br>0.014<br>(0.008-0.033)	n=11<br>0.012<br>(0.003-0.020)	NA

Values are reported as M (IQR), median and interquartile range. Calculations were made including results falling in the quantification range as detailed in the methods section. All biomarker values are normalized to urine-creatinine levels, i.e. expressed as weight units per mmol of urine creatinine.

Supplementary Table 3. Clinical characteristics and u-Gal-3BP concentration in patients with repeated biopsy before and after induction treatment

**Patient**	**Gender/Age**	**S-creatinine**	**Treatment before and after first biopsy**		**ISN baseline and at repeated biopsy**		**u-Gal-3BP (ng/mmol of creatinine) at first and second biopsy**	
**1**	F/41	145	0	CYC	IV°	IV	102411	6622
**2**	M/18	51	MTX	MMF	V	III (A)+V	13558	15987
**3**	F/42	73	0	MMF	III (A) +V	V	38516	15991
**4**	F/35	49	0	MMF	III (A) +V	I	23699	NA^
**5**	F/33	MD	0	CYC	V	V	5427	14603
**6**	F/52	80	0	CYC	IV-S(A)	II	6798	2108
**7**	F/33	76	0	MMF	IV-G(A)	IV-G (A) + V	23420	6371

					+V			
**8**	F/22	104	0	CYC/ MMF	IV-G(A) /+V	IV-G(A) /+V	445593	15591
**9**	F/61	154	0	RTX +CYC	IV-S(A/C)		4698	1229
**10**	F/52	173	0	RTX	V		16388	8836
**Median (IQR)**							19904 (6455-54490)*	7729 (1888-15690)*

*Wilcoxon signed rank test *p=0.03*

° classified according to WHO

^follow-up u-Gal-3BP concentration under the detection limit
